# Influence of Flow Disturbances behind the 90° Bend on the Indications of the Ultrasonic Flow Meter with Clamp-On Sensors on Pipelines

**DOI:** 10.3390/s21030868

**Published:** 2021-01-28

**Authors:** Piotr Synowiec, Artur Andruszkiewicz, Wiesław Wędrychowicz, Piotr Piechota, Elżbieta Wróblewska

**Affiliations:** Faculty of Mechanical and Power Engineering, Department of Thermal Science, Wrocław University of Science and Technology, Wybrzeże Wyspiańskiego 27, 50-370 Wrocław, Poland; artur.andruszkiewicz@pwr.edu.pl (A.A.); wieslaw.wedrychowicz@pwr.edu.pl (W.W.); piotr.piechota@pwr.edu.pl (P.P.); e.wroblewska@pwr.edu.pl (E.W.)

**Keywords:** balancing, flow velocity, ultrasonic flow meter, laser Doppler anemometer, LDA

## Abstract

The subject matter of the article concerns velocities/flow rate measurements in the area of disturbed flows-behind the 90° bend. They were conducted by means of an ultrasonic flowmeter with clamp-on sensors on pipeline, for water and two different Reynolds numbers of 70,000 and 100,000, corresponding to two velocities of approximately 1.42 m/s and 2.04 m/s. The tests were carried out at 12 distances from the disturbance. Sensors on the circumference of the pipeline were mounted 30° each. The correction factor values were calculated for the given measurement geometry. The measurements have shown that the values of this coefficient are always greater than 1, which means that the ultrasonic flow meter understates the speed values. They also showed that already at a distance of 8 nominal diameters from the disturbance, the correction factor does not exceed 1.02, so the measurement errors are within the maximum permissible error (MPE) of a typical ultrasonic flow meter. For distances less than eight nominal diameters from the disturbance, not taking the correction factor value into the account can lead to systematic errors of up to 10.8%. Studies have also proved that in each measurement plane behind the disturbance there are two mounting angles for the ultrasonic sensors, 60° and 240° respectively, for which the correction factor values are minimal. Additionally, using the laser Doppler anemometry (LDA) method, velocity solids were determined at individual distances from the disturbance, and the projections of velocity blocks on the appropriate plane represented velocity profiles and indicated the distances from the disturbance at which these profiles stabilise.

## 1. Introduction

Performing the correct measurement of the stream flow of the working medium in industrial hydraulic installations is one of the most important and most difficult things to achieve. It is related to many factors influencing measurement processes, such as geometrical distortion of pipelines with circular cross-section, sediment inside the pipelines [1,2,3,4], elements of fittings disturbing the velocity profile and temperature of the measured medium. Modern development of technical culture and measurement technique forces making measurements as accurately as possible, due to the fact that measurement information is used for various purposes [5,6]. An exemplary goal is to use the data to control technological processes in chemical, petrochemical, municipal water supply industries as well as in power engineering installations. In case of chemical installations, the use of accurate measurements is associated with maintaining the appropriate stoichiometry of the processes. In waterworks, pump efficiency measurements are performed and water consumption by consumers is determined, both instantaneous and average.

Commercial power industry, which includes power plants, electrical power and heating plants and heating plants, uses flow meters to balance internal and external water management. Such action is aimed at determining possible losses in the installation, which is associated with the need to prepare and supplement the medium in the installation. In this case, the measuring accuracy of the devices should be as high as possible, assuming the lowest possible investment costs. That is why in each case of measurement, the most important factor is the selection of the best available measurement technique in relation to costs [7,8,9]. Performing flow measurements is characterized by a multitude of solutions, such as Pitot tubes, measuring orifices, turbine flow meters, bend flow meters, vortex flow meters, Coriolis flow meters, electromagnetic flow meters and ultrasonic flow meters [10,11,12,13,14].

The non-invasive ultrasonic technique is an increasingly popular measurement technique allowing to determine the velocity of a fluid with a maximum permissible error (MPE) not exceeding 2% of the indicated value. This accuracy is ensured in case of long measuring sections exceeding 15 pipeline diameters in which the distribution of fluid velocity in the cross-section is fully developed. An element of every industrial installation are gate valve elbows or valves which distort the fluid velocity profiles. In the absence of sufficiently long straight sections, measurements are made at a small distance from these elements, which causes additional measurement errors. The question therefore arises as to what are the values of these errors and whether they can be reduced by an appropriate location of ultrasonic factors in the measuring section.

Research on the influence of the velocity profile on the accuracy of ultrasonic flow meters (Doppler and Transit-Time type) measurements is carried out in different research institutions involved in the issue of flows. Measurements are carried out on long straight sections where the velocity profile changes with an increase in the Reynolds number, as well as behind typical disturbing elements: bends, double bends, constrictions or valves. In the first case Zhang et al. [6] calculated the correction factor values and measurement errors up to a maximum Reynolds number of 422,122. In the second case the work of Wada and Furuichi [15] investigating the influence of obstruction plates on the uncertainty of flow rate measurement based on the velocity profile method with Doppler ultrasonic method as well as the work of Treenusonn et al. [16] investigating the influence of double knees on the accuracy of Doppler flow meters should be mentioned. An article by Masasi [17] who, after Eisenhauer [18], gave the values of correction coefficients at various distances behind a 90° bend in Transit-Time flowmeter measurements, although without specifying how they were averaged, is also interesting. Errors of this type of flowmeter measurement behind a 45° bend were also presented by Srith et al. [19].

The purpose of this article is to determine the measurement errors of velocity (flow rate) behind the 90° bend for two different flow rates and for 12 positions (every 30°) of the ultrasonic sensors in a given measurement area and at different distances from the disturbance. Such small measurements also indicate the place of sensors installation on the perimeter of the pipeline for which the measurement error will be the smallest. Additionally, in comparison with other works on this subject [20,21,22,23,24], using laser anemometry methods, velocity lumps were determined at particular distances from the disturbance, and velocity projections from those lumps on the appropriate plane represented velocity profiles and indicated the distances from the disturbance in which those profiles stabilize. 90° bend was selected because it is the most common element of the flow system, whereas in ultrasound measurements method of two ultrasound wave transitions (V method) was used, also the most frequently performed in the measurement practice [25,26,27,28,29].

## 2. Measurements

### Measurement Stand

**Figure 1:**
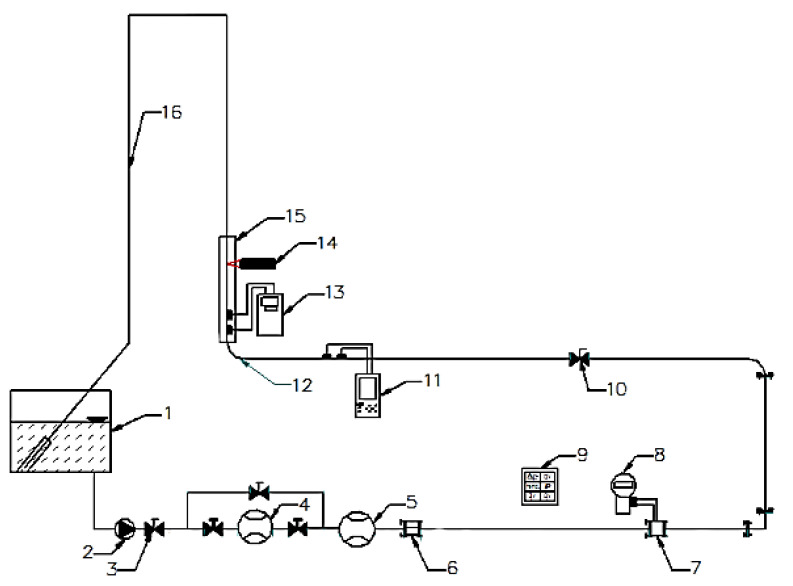
Experimental stand. 1—container with water; 2—pump; 3—control valve; 4—coupled water meter; 5—electromagnetic flow meter; 6—stream straightener; 7—ISA orifice with annular chamber pressure tapping; 8—pressure transducer; 9—programmable current recorder; 10—throttle; 11—reference ultrasonic flow meter measuring in an undisturbed area; 12—90° bend with radius R = 2D; 13—ultrasonic flow meter measuring in an area of disturbed flow; 14—LDA laser Doppler anemometer; 15—correction vessel; 16—return recirculator pipeline. Description in the text.

In order to carry out research on the accuracy of ultrasonic measurements which were performed in the area of disturbed flow occurring behind the 90° bend, a special measurement stand–shown in the diagram below–was designed and constructed (Figure 1). The stand includes a container with water (1), a pump (2), a control valve (3), a throttle (10), a distorting element which is the 90° bend (12) with radius R = 2D and installation pipes (16) with the inner diameter of 50mm. To achieve high accuracy of measurements of the reference flow stream several flow meters taking the measurements simultaneously were used. Coupled water meter (4) measuring water volume and time, electromagnetic flow meter (5) and ISA orifice with annular chamber pressure tapping (7) were used. To assure the correctness of measurement made with the orifice stream straightener (6) was also used. Measurement data from the flow meters as well as from the pressure transducer (8) of the orifice were recorded with the recorder (9) having programmable entry zones which enable adjusting and recording every kind of signal from the flow meters. A temperature sensor was also connected to the recorder and on the basis of measured temperature and the flow stream the recorder calculated the Reynolds number. The use of several different flow meters allowed for the improvement of accuracy of the reference flow and the Reynolds number as a criterion of reconstructing measurement conditions influenced the accuracy of the results obtained [1,2,3,4].

Because the purpose of the measurements was to set the influence of the distortion of the velocity profile on the indications of the ultrasonic flow meter on the pipeline the ultrasonic flow meter (11) was mounted prior to the distortion with full compliance with the requirements specified by the producer to achieve the correct measurement. This flow meter was of the same type and model as the flow meter on which the research was conducted (13) in the distorted area. Both of the flow meters had clamp-on heads placed on the pipeline.

For better understanding of the phenomena occurring during the measurement in the distorted area and correct interpretation of the results of measurements made with ultrasounds, a laser Doppler anemometer (LDA) was used to determine an actual velocity profile of the pipeline cross-section. To make the non-invasive LDA measurement possible measurement sections of the pipeline were made of organic glass and surrounded with a correction vessel for the time of measurements to eliminate the effect of refraction of laser light on the curvilinear surface. The results obtained from the LDA measurements in the form of velocity profile will be used for experimental verification of CFD modelling of the flow stream in the tested distortion [5,6,7].

In Table 1 and Table 2 technical data of equipment as well as measurement settings are presented.

## 3. Experiment

The research was conducted on a measurement stand presented on Figure 1. To maintain recurrence of the measurement conditions the Reynolds number was taken as a criterion. The Reynolds number was computed in real time on the basis of temperature and volume stream data measured by the recorder with mathematical operations function (element 9). The measurements consisted in simultaneous measuring of the flow stream with different types of flow meters (elements 4, 5, 7, 8, 11) to determine the real flow with the highest possible accuracy, the flow with a referential ultrasonic flow meter (element 11) in the area of a non-distorted flow as well as ultrasonic flow meter of the same type as the referential one in the distorted area (element 13) were measured.

**Figure 2:**
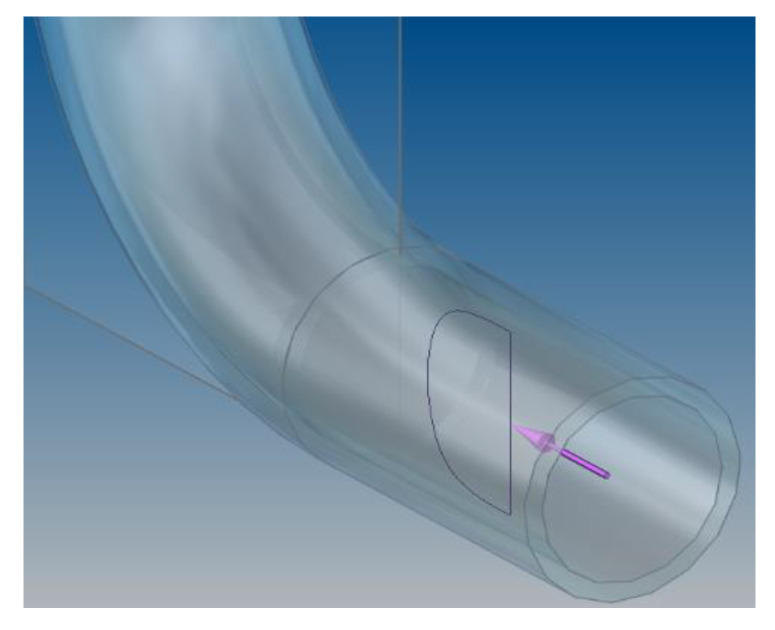
0-degree dividing plane.

The ultrasonic beam of the flow meter determines the average velocity of the fluid in one plane passing through the diameter of the pipe. Considering the possibility of an asymmetrical velocity distribution in a cross-section of the pipe in the distorted area the measurements were made in 6 different planes passing through the diameters shifted every 30 degrees (whereby every diameter was measured twice so that the head transmitting the ultrasonic beams was changing its position every 30 degrees in the 360 degree range) for every distance from the obstacle. In the description of results it was assumed that the initial angle 0 degrees will be positioned in the inner side of the 90° bend. On Figure 2 the plane passing through the 90° bend for 0 and 180 degrees was presented.

**Figure 3:**
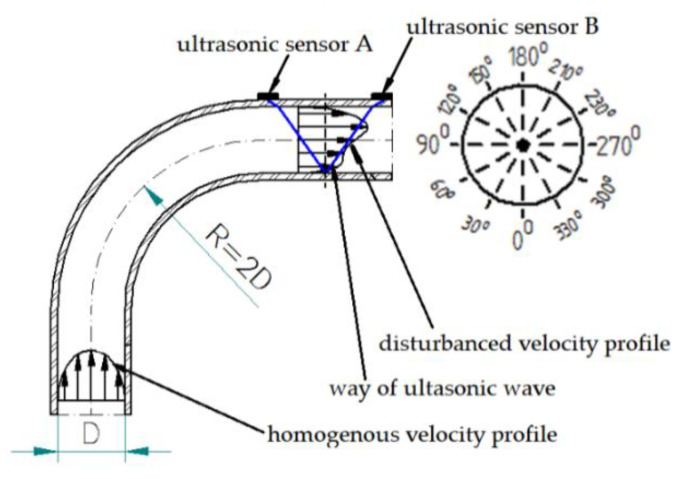
Diagram of the measurement area.

Because of the change of the distorted velocity profile which occurred with the change of the distance from the obstacle the measurements were made in the distances defined by the multiplicity of the diameter of the pipeline. The measure closest to the obstacle was indicated as 0D and next measurements every 1 diameter until 15 diameters were reached. The series of measurements were taken for selected Reynolds numbers of 70,000 and 100,000, determined by the capability of the stand. The average flow velocity with these Reynolds numbers was indicated in the range 1.418–2.04 m/s which is a typical velocity used in pipelines. Because of a small diameter of the experimental pipeline the heads of the ultrasonic flow meter were mounted in a V-system as it shown on Figure 3. This figure also shows the angles for which the measurements were taken.

For researches on the structure of the stream of flow with a distortion, the non-invasive laser anemometry measurements of the velocity distribution were used. In selected cross-sections, in which the measurements with the ultrasonic flow meter were conducted, measurements of the velocity in the whole cross-section of the pipe were made with a laser anemometer. The measuring points were put on a square grid with 2 mm pitch. To keep the distance between the measurement points within the pipe cross-section constant shifts connected to the refractions of the ray on the border between two media was included. Due to the refraction of light the intersection of laser beams changes its position which determines the necessity of correction of the head shift. To improve the conditions of measurement a seeding of finely ground mica, which is light and moves with the fluid and strongly reflects light at the same time, was used in the installation. To prevent settling of the seeding inside of the tank, water was periodically stirred.

On the basis of single topical velocities the velocity blocks were prepared for the pipe cross-sections, which are the basis of further analysis.

## 4. Experimental Results

The results of ultrasonic measurements and laser Doppler anemometry (LDA) researches for Reynolds criterion numbers Re = 70,000 and Re = 100,000 will be presented and discussed:

Figures 5, 11 and 21 show the velocity obtained at different distances from the element disturbing the flow. Flow parameters were described by the Reynolds criterion number equal approximately 70,000, which proves the turbulent nature of the phenomenon.

Figures 6, 12 and 22 show, similarly to the previous ones, velocity blocks obtained at different distances for the Reynolds number equal approximately 100,000. Thanks to laser Doppler anemometry, it is possible to study flow phenomena occurring for example behind various elements of industrial hydraulic installations. The anemometric tests allow to know the complexity of phenomena occurring behind the flow disturbing element. They also allow for industrial measurements to determine correction coefficients for a given angle of installation of ultrasonic heads in the area of disturbed flow.

**Figure 4:**
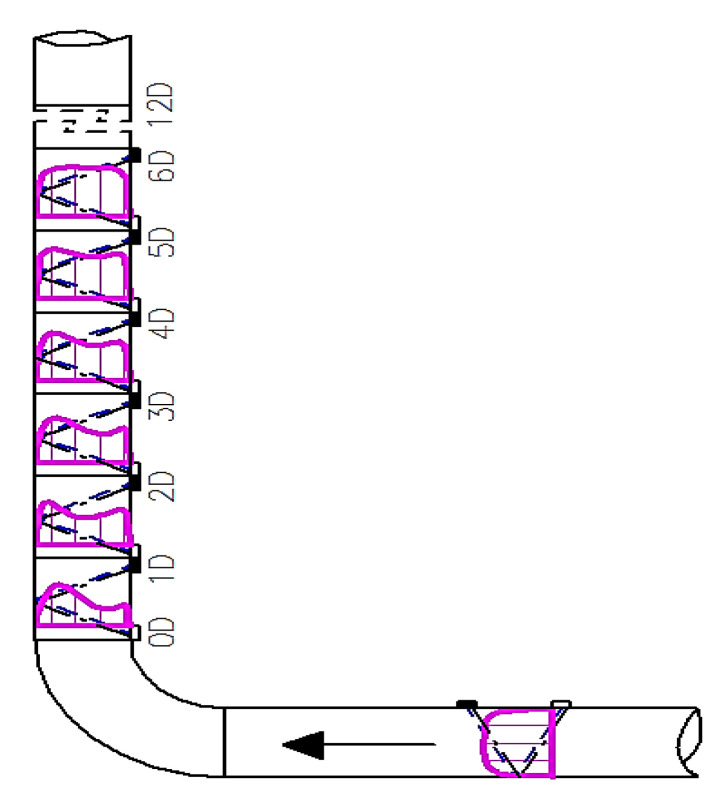
Diagram of the distribution of measuring cross-sections in a flow system.

**Figure 5:**
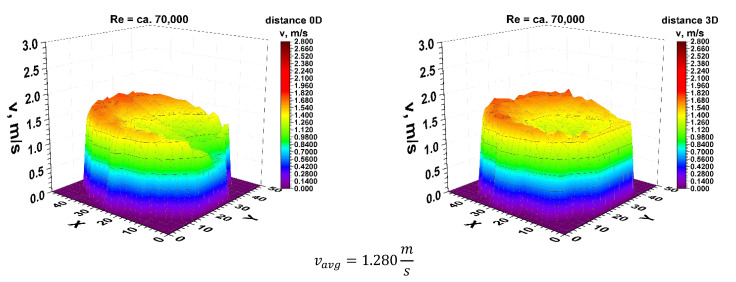
Comparison of velocity blocks at the distances 0D and 3D from the disturbance obtained by means of LDA measurements for Re ≅ 70,000.

**Figure 6:**
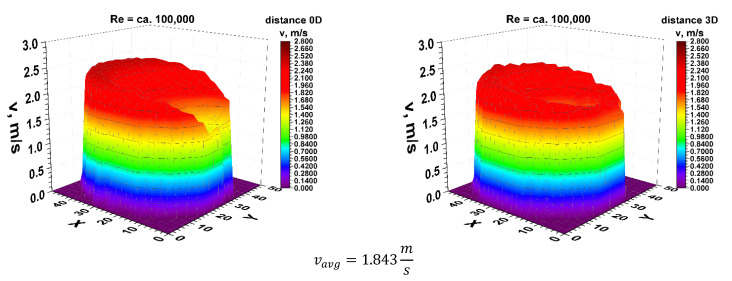
Comparison of velocity blocks at the distance 0D and 3D from the disturbance obtained by means of LDA measurements for Re ≅ 100,000.

First, 3D velocity distribution were made in order to qualitatively and quantitatively check the changes taking place in the velocity profile at different distances from the disturbing element, as shown in Figure 4. Figure 5 and Figure 6 show the results obtained from the plane directly behind the 90° bend and defined as the distance 0D, i.e., zero nominal pipeline diameters from the element causing disturbances in the flow of the measured medium.

It can be concluded from the figures presented in diagrams 5 and 6, which are the real reflection of the geometry of the velocity profile directly behind the 90° bend, it can be seen that by making a qualitative comparison they are similar to each other. There is a recess in the 3D velocity distribution at the angle of water inflow to the pipeline bend. These recesses prove that in these places the flow velocity of the measured medium is lower on the inside of the pipeline.

**Figure 7:**
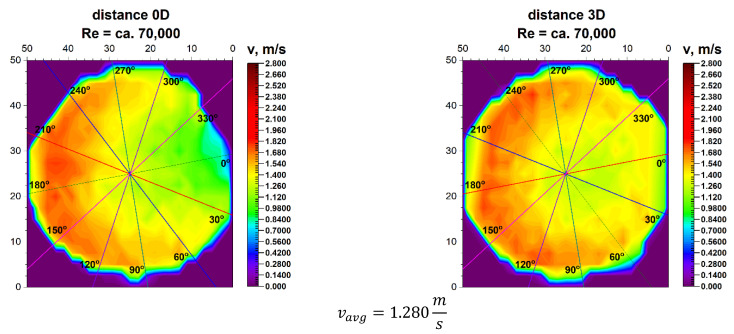
Comparison of projection on the velocity blocks at a distances 0D and 3D from the disturbance with marked cross-sections for Re ≅ 70,000.

**Figure 8:**
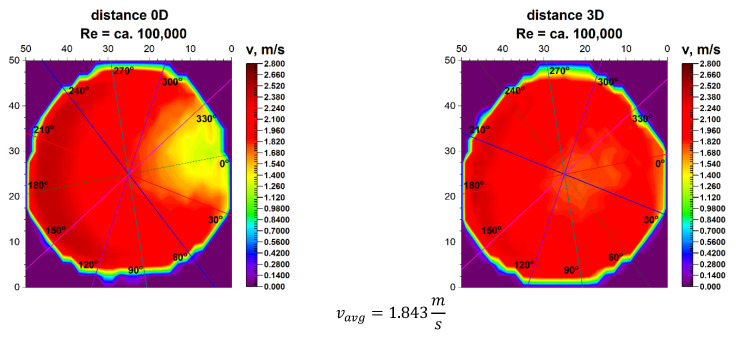
Comparison of projection on the velocity block at the distances 0D and 3D from the disturbance with marked cross-sections for Re ≅ 100,000.

The velocity blocks obtained for the various Reynolds numbers, illustrated in Figure 5 and Figure 6, are projected onto the plane and shown in Figure 7 and Figure 8. The lines on the projection mark the angles for which flow measurements were carried out with the use of ultrasonic flow meters with heads placed on the pipeline.

The initial angle marked as 0° is the inflow angle to the bend. This angle is on the axis of the pipeline and is also the symmetry plane of the element disturbing the flow. The projections in Figure 7 and Figure 8 accurately show the distribution of velocity contours in the cross section immediately behind the 90° bend. This distance is defined as 0D, which defines the distance between the measurement plane and the disturbing element expressed in nominal pipeline diameters.

In the LDA graphs No. 7, 8, 13, 14, 19, 23, 24 the areas with the highest liquid flow velocities are marked with red and its shades, while the areas with no flow are marked with dark blue.

In both described graphs for the flow zone characterized by the Reynolds number ≅ 100,000 as well as for the zone where the Reynolds number ≅ 70,000, a semicircular distribution of the highest velocities in the outer part of the pipeline is noticeable, which indicates the system inertia and the fact that on the inside of the pipeline there may be vortexes. Such assumptions have been presented in the literature on fluid mechanics [30], however, it applies only to the classic bend.

For technical reasons, the measurement with the laser anemometer was performed only for the X component of the velocity of the measured medium, which is the equivalent of measuring with a Prandtl tube. After calculating the integrals of the surfaces obtained as a result of measurements of the 3D velocity distribution obtained by laser anemometry, the results were obtained v_avg_ = 1.843 m/s for the flow where Re ≅ 100,000 and v_avg_ = 1.280 m/s for the flow where Re ≅ 70,000.

**Figure 9:**
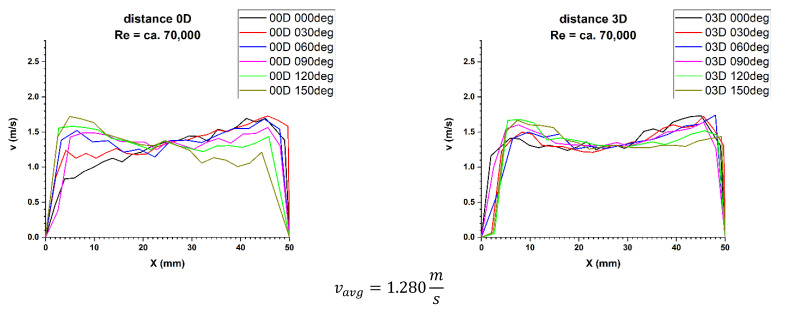
Comparison of velocity profiles at a distances 0D and 3D from the disturbance with marked cross-sections for Re ≅ 70,000-The LDA measurement taken in the ultrasound wave path.

**Figure 10:**
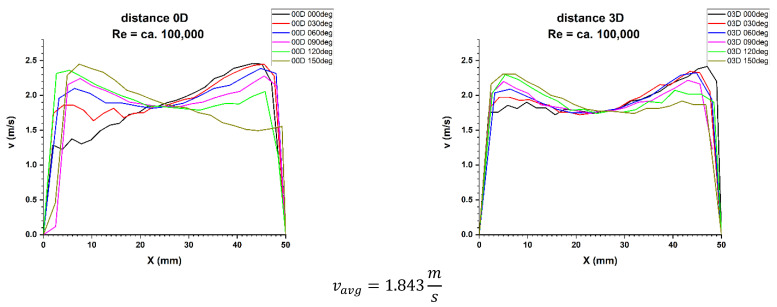
Comparison of velocity profiles at a distances 0D and 3D from the disturbance with marked cross-sections for Re ≅ 100,000-The LDA measurement taken in the ultrasound wave path.

Figure 9 and Figure 10 show the cross-sections of 2D velocity distribution for the 0D plane at individual mounting angles of the ultrasonic flow meter heads. It is also noticeable that the profiles in terms of quality are similar to each other and intersect in the pipeline axis, which proves the correctness of the conducted measurement.

From the results presented in Table 3 it can be seen that for the Reynolds number ≅ 70,000 and the plane located directly behind the 90° bend there are installation angles of the ultrasound heads where the measurement errors are the smallest. For angles of 60° and 240° measurement errors are 7.9% and 8.6%. There is also a noticeable difference Δv_avg_ which equals to 6.2% between the minimum and maximum of the measurements taken behind the 90° bend in the 0D plane.

The mean correction factor K^*^v_avg_ is 10.8%. When multiplying the obtained results by a coefficient reflecting Δv_avg_, which is 1.061, the measurement at an angle of 60° is characterized by the smallest error, which is within the maximum permissible error (MPE) of the ultrasonic flow meters equal to ±2%. The coefficient 1.061 is the ratio v_avg max_ and v_avg min_. The remaining results are beyond the maximum permissible error (MPE) of the flow meters. Therefore, it should be concluded that the measurements made in the 0D plane, with Reynolds number ≅ 70,000, should be accompanied by an average correction factor of 1.108 in order to minimize the uncertainty of the measurements.

It can be seen from Table 4 that for the Reynolds number ≅ 100,000 and the plane directly behind the 90° bend, there are two angles of the ultrasonic head installation for which the measurement error is the smallest. For angles of 60° and 240° it is 5.6%. There is also a noticeable difference Δv_avg_ which equals to 8% between the minimum and maximum of the measurements made behind the 90° bend in the 0D plane. The mean correction factor K^*^v_avg_ is 10.8%. When multiplying the obtained results by a coefficient reflecting Δv_avg_, which is 1.08 the measurements taken at the angles of 30° and 90° have the smallest error which is within the maximum permissible error (MPE) of the ultrasonic flow meters and equals to ±2%.

The remaining results are beyond the maximum permissible error (MPE) of the flow meters. Therefore, it should be concluded that the measurements made in the 0D plane, with Reynolds number ≅ 100,000, should be accompanied by an average correction factor of 1.108 in order to minimize the uncertainty of the measurements.

**Figure 11:**
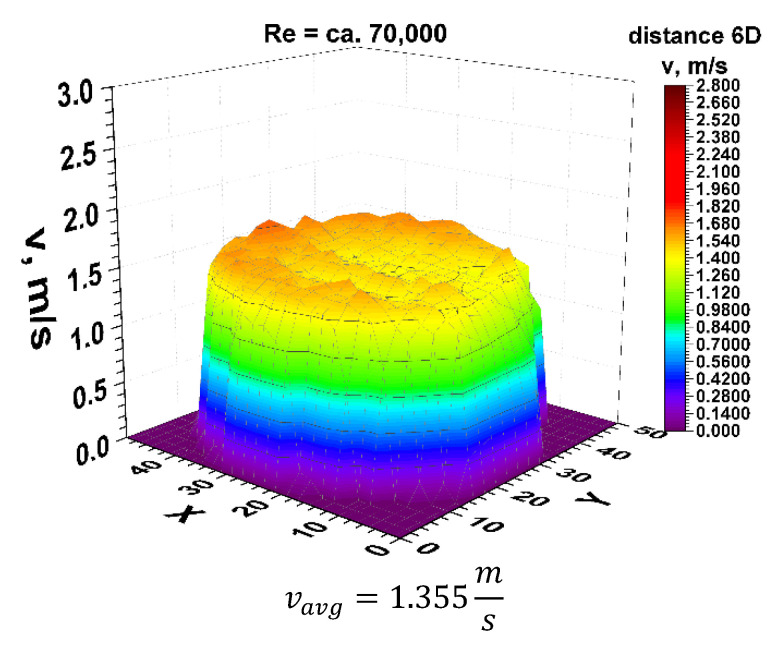
Velocity block at the distance 6D from the disturbance obtained by means of LDA measurements for Re ≅ 70,000.

**Figure 12:**
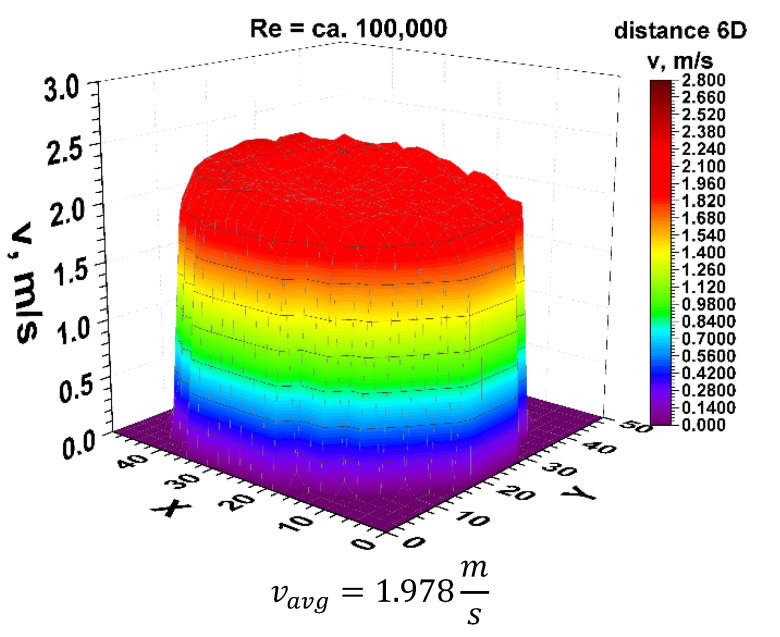
Velocity block at the distance 6D from the disturbance obtained by means of LDA measurements for Re ≅ 100,000.

Going to the distance indicated by 6D, the spatial velocity profiles for the two different volume streams shown in Figure 11 and Figure 12 clearly have a closed parietal area on the side corresponding to the inside of the 90° bend. However, in the 3D velocity distribution, you can notice a clear disappearance of the recess near the pipeline axis.

**Figure 13:**
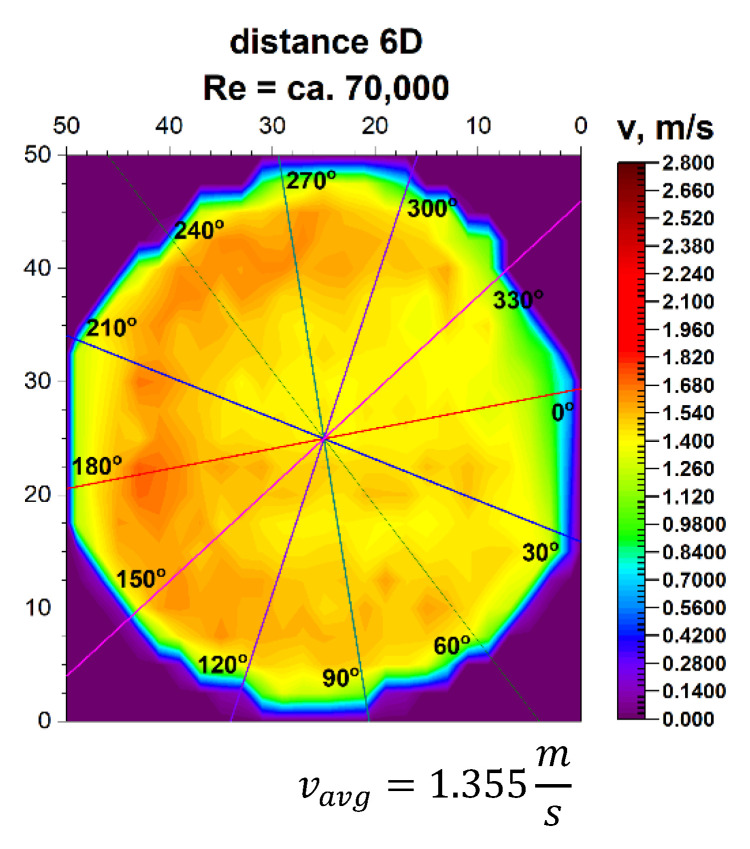
Projection on the velocity block the distance 6D from the disturbance with marked cross-sections for Re ≅ 70,000.

**Figure 14:**
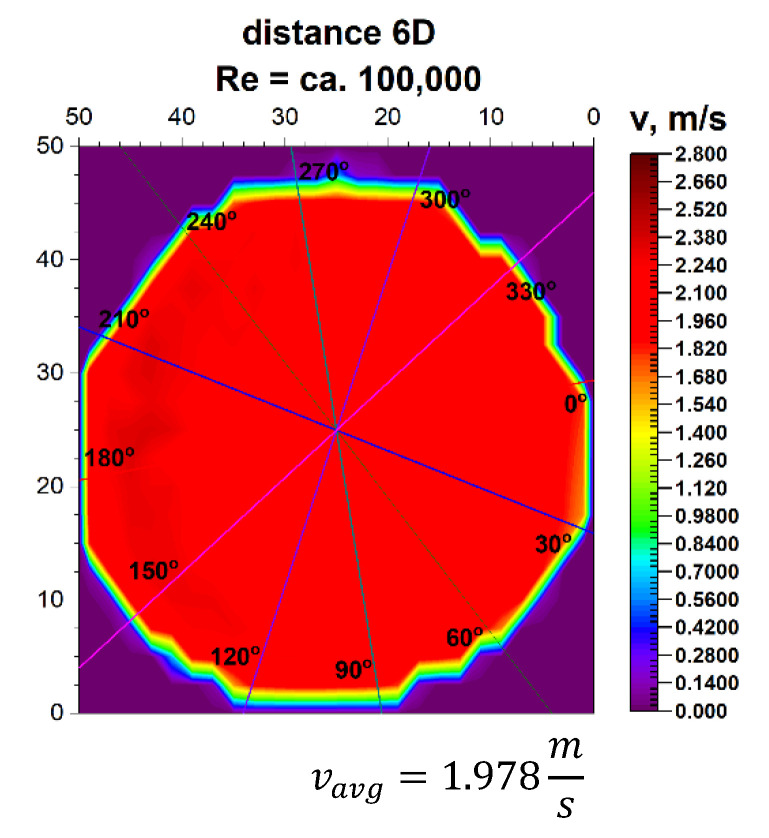
Projection on the velocity block at the distance 6D from the disturbance with marked cross-sections for Re ≅ 100,000.

The stabilization of the 3D velocity distribution is noticeable. The obtained results for various Reynolds numbers, shown in Figure 11 and Figure 12, were projected onto the plane, which is shown in Figure 13 and Figure 14, where, analogously to the distance 0D, cross-sections in accordance with the mounting angles of the ultrasonic heads were made. The above graphs show that the velocity profiles begin to become flat in the central part of the 3D velocity distribution, which indicates the stabilization phase of the flow.

The plane projections on the 3D velocity distribution at a distance of 6D from the 90° bend shown in Figure 13 and Figure 14 clearly illustrate the behaviour of the liquid behind the disturbing element with increasing distance from the disturbing element. In the described projections, especially in the visualization of the flow corresponding to the Reynolds number ≅ 100,000, one can observe the vanishing area with the highest flow velocity, which in the previous profiles was located along the outer wall of the pipeline corresponding to the outer part of the bend.

The flow velocity contours no longer appear along the bend, which may indicate a stable nature of the flow with increasing distance behind the 90° bend. Also, in the flow corresponding to the Reynolds number ≅ 70,000, the area with the highest flow velocity is located along the outer wall of the pipeline, with a clear emphasis on the increase in velocity in the remaining parietal areas of the pipeline, which may be evidenced by the uniformity of the green color in the diagram.

Both graphs show an increase in flow velocity in the center of the pipeline. In both cases, the above mentioned indicates a probable stabilization of the velocity profile. In this situation, it can be concluded that it is probably possible to measure the flow stream at a distance of six nominal diameters from the 90° bend, with the metrological parameters declared by the manufacturer of the measuring device.

After calculating the integrals from the surfaces obtained as a result of measurements of 3D velocity distribution obtained by laser anemometry, the results were obtained as an average velocity module v_avg_ = 1.978 m/s for the flow, where Re ≅ 100,000 and v_avg_ = 1.355 m/s for the flow, where Re≅70,000.

**Figure 15:**
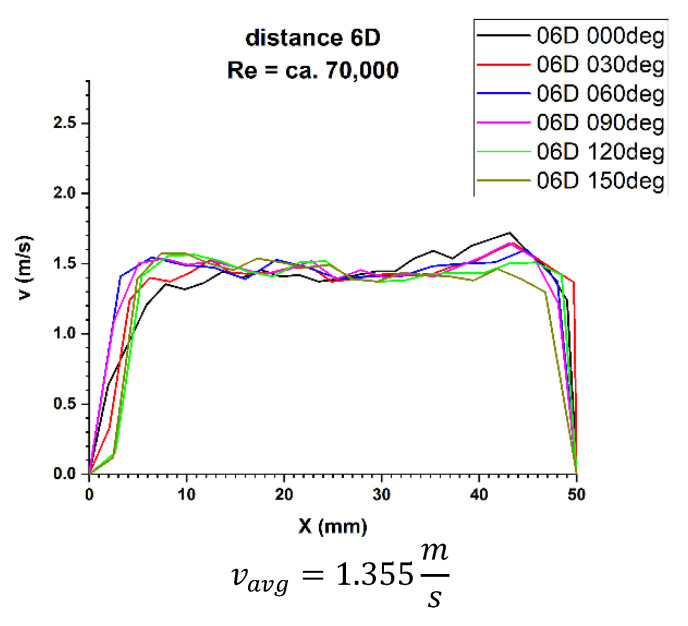
Velocity profiles at a distance 6D from the disturbance with marked cross-sections for Re ≅ 70,000-The LDA measurement taken in the ultrasound wave path.

**Figure 16:**
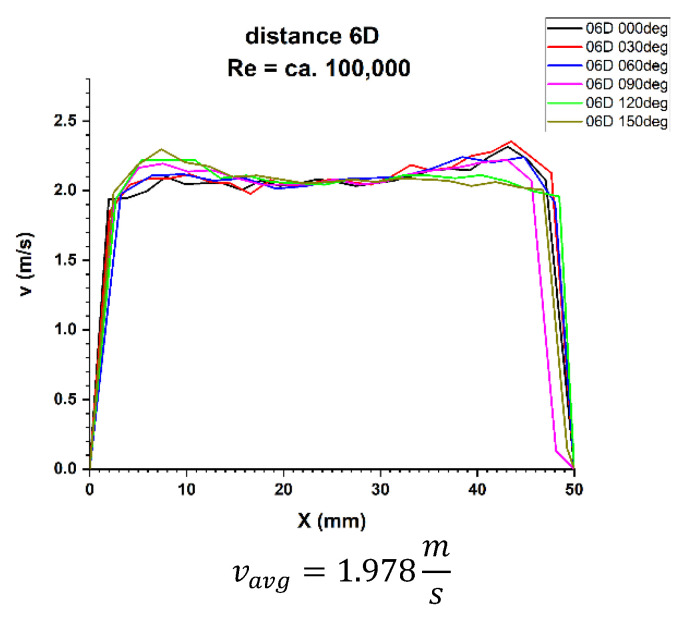
Velocity profiles at a distance 6D from the disturbance with marked cross-sections for Re ≅ 100,000-The LDA measurement taken in the ultrasound wave path.

In the velocity profiles for the plane 6D of the pipeline diameters behind the 90° bend, the minimum and maximum velocities are clearly noticeable, which proves the non-homogeneous flow. It is also noticeable that the profiles are similar in terms of quality. The profiles shown in Figure 15 and Figure 16 intersect along the pipeline axis, which proves that the measurement was performed correctly.

In the graphs 16 and 17 for the 6D plane, showing the two-dimensional velocity profiles, it can be seen that both in a flow with a Reynolds number of ≅100,000 and in a flow corresponding to a Reynolds number of ≅70,000, the shapes of the profiles at individual angles are similar.

In both cases, velocity minima in the surroundings of the pipeline axis combined with the flatness of the profile in the central part of the pipeline can be seen. First of all, in both cases the profile closure corresponding to the 0° angle in the inflow zone was observed, as well as the convergence of the profiles in relation to each other. The stability of the velocity profile can be qualitatively determined.

In Table 5 it can be seen that for the Reynolds number ≅ 70,000 and the plane located at a distance 6D behind the 90° bend, there are two installation angles of the ultrasound heads where the errors of the direct measurements are the smallest. For angles of 60° and 240° they are 3.5% and 3.2%, respectively. There is also a noticeable difference Δv_avg_ equal 2.9% between the minimum and maximum of the measurements made behind the 90° bend in the 6D plane. The mean correction factor K^*^v_avg_ is 4.6%, so it should be concluded that the measurements made in the 6D plane, with the Reynolds number about 70,000, should have an average correction factor equal 1.046 in order to minimize the uncertainty of the measurements.

In Table 6 You can see that for the Reynolds number ≅ 100,000 and the plane located at a distance 6D behind the 90° bend, there are two installation angles of the ultrasound heads where the errors of the direct measurements are the smallest. For angles of 60° and 240° it is 2.2%. There is also a noticeable difference Δv_avg_ equal 1.7% between the minimum and maximum of the measurements made behind the 90° bend in the 6D plane. The mean correction factor K^*^v_avg_ is 3.1%. So it should be concluded that the measurements made in the 6D plane, with Reynolds number ≅ 100,000, should have an average correction factor equal 1.031 in order to minimize the uncertainty of the measurements.

Results of research carried out at distances of 8D and 10D from the flow disturbing element, where the homogenisation of the velocity profile was found, are presented in Table 7 and Table 8.

**Figure 17:**
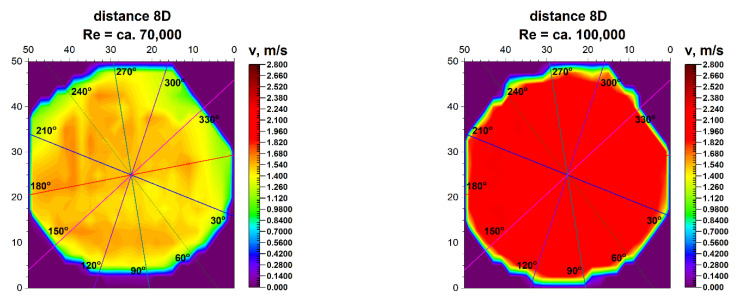
Comparison of projections on the velocity blocks the distances 8D from the disturbance with marked cross-sections for Re ≅ 70,000 and 100,000.

**Figure 18:**
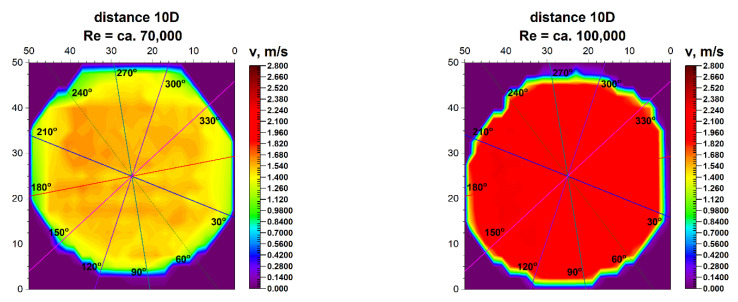
Comparison of projections on the velocity blocks the distances 10D from the disturbance with marked cross-sections for Re ≅ 70,000 and 100,000.

Figure 17 and Figure 18 compare the velocity distributions in 8D and 10D from the 90° bend.

**Figure 19:**
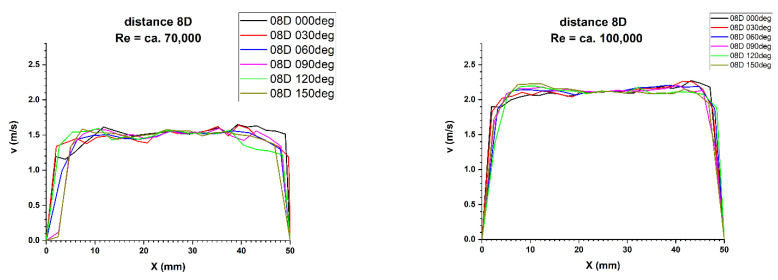
Comparison of velocity profiles at a distance 8D from the disturbance with marked cross-sections for Re ≅ 70,000 and 100,000-The LDA measurement taken in the ultrasound wave path.

**Figure 20:**
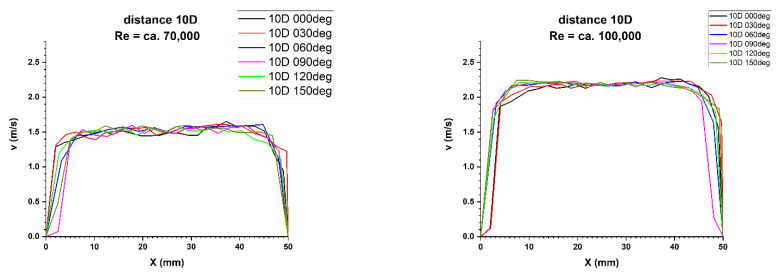
Comparison of velocity profiles at a distance 10D from the disturbance with marked cross-sections for Re ≅ 70,000 and 100,000-The LDA measurement taken in the ultrasound wave path.

Figure 19 and Figure 20 illustrate the comparison of velocity profiles obtained by laser Doppler anemometry at distances of 8D and 10Dalong the path of the ultrasonic wave. That allows to clearly illustrate the nature of the obtained results.

**Figure 21:**
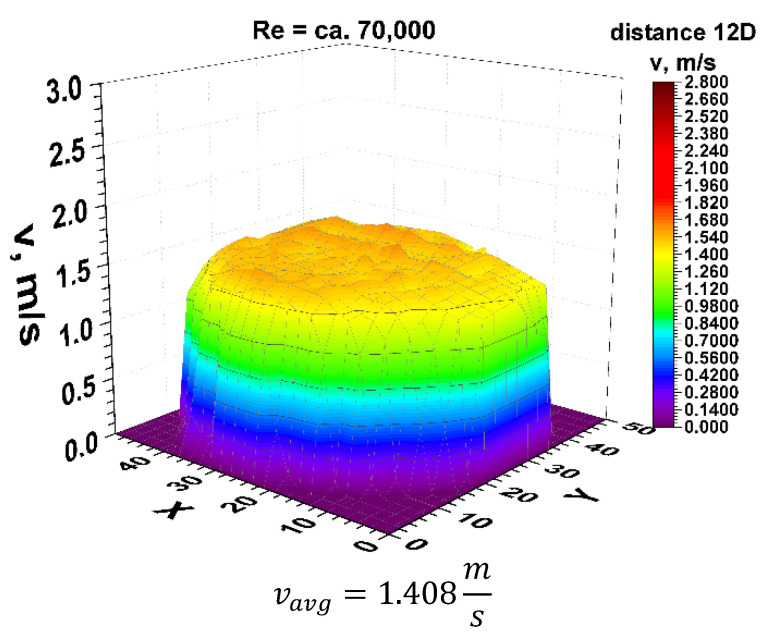
Velocity block at the distance 12D from the disturbance obtained by means of LDA measurements for Re ≅ 70,000.

**Figure 22:**
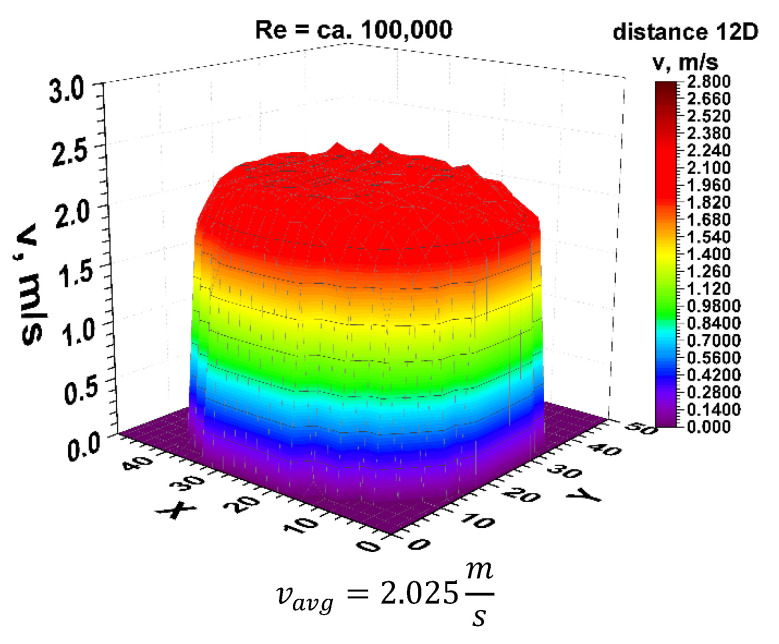
Velocity block at the distance 12D from the disturbance obtained by means of LDA measurements for Re ≅ 100,000.

Finally, the focus should be on the distance of 12 nominal pipeline diameters from the disturbance. Figure 21 and Figure 22 show the 3D velocity distribution for two different volume streams, expressed with Reynolds criterion numbers. In a plane separated by twelve nominal pipeline diameters, represented by the marking 12D, they have a characteristic 3D velocity distribution. 3D velocity distribution for both streams clearly shows that the velocity profile has stabilized [1,2] The above graphs show that the velocity profiles are flat in the central part of the 3D distribution, which indicates that the flow velocity profile is stabilized.

From the graphs in Figure 21 and Figure 22, reflecting the geometry of the velocity profile at a distance of 12D behind the 90° bend, it can be concluded that they are similar to each other. Stabilization of the 3D velocity distribution is noticeable. The obtained results for different Reynolds numbers, presented in Figure 11 and Figure 12, were projected onto the plane, which is shown in Figure 13 and Figure 14, and, similarly to the distances 0D and 6D, cross-sections were made in accordance with the mounting angles of the ultrasonic heads.

**Figure 23:**
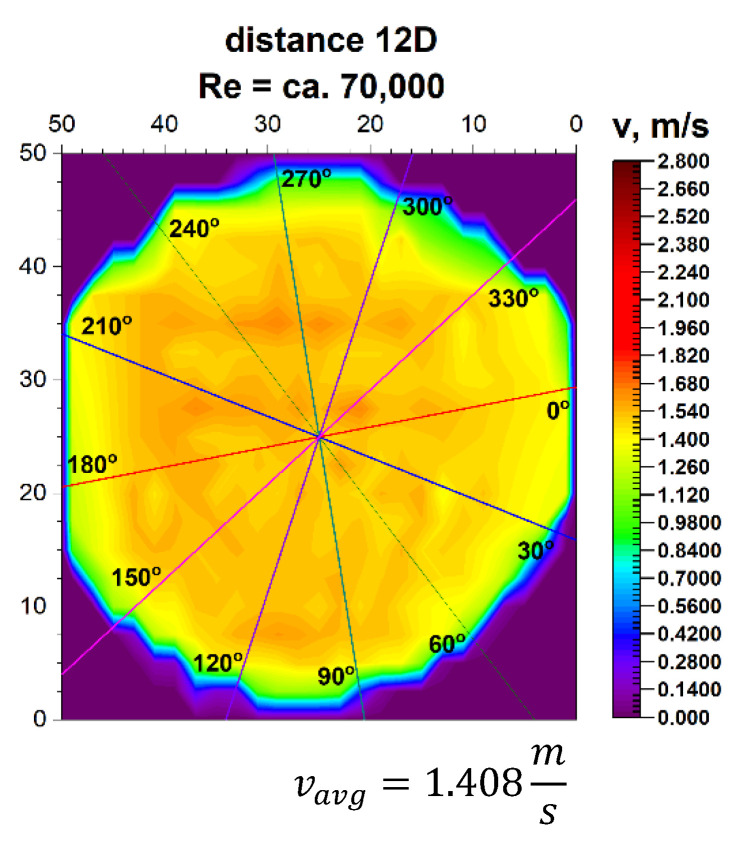
Projection on the velocity block at the distance 12D from the disturbance with marked cross-sections for ≅ 70,000.

**Figure 24:**
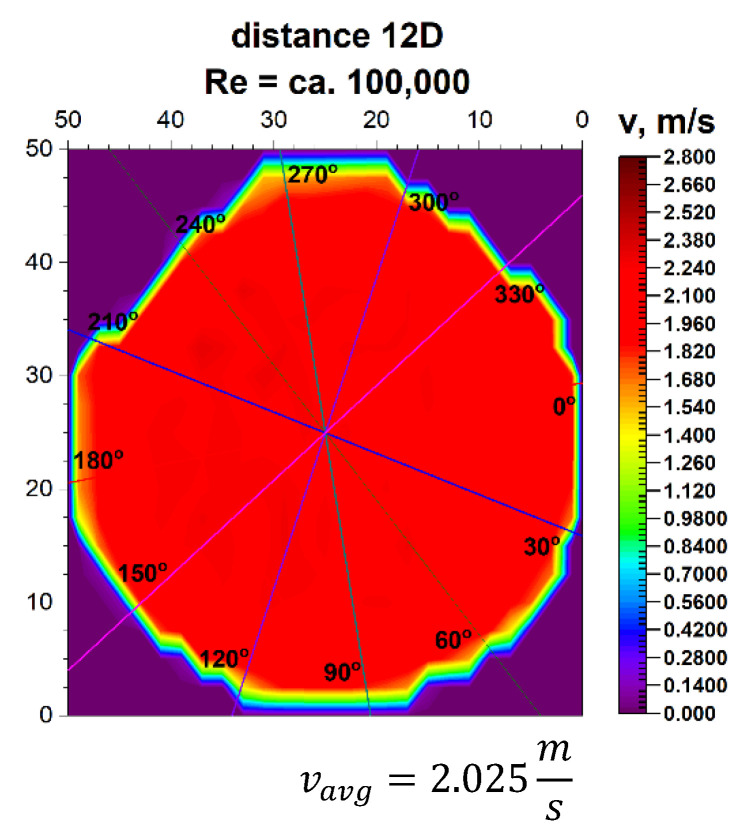
Projection on the velocity block at the distance 12D from the disturbance with marked cross-sections for Re ≅ 100,000.

The plane projections on the 3D velocity distribution occurring at a distance of 12D from the 90° bend, presented in Figure 23 and Figure 24, clearly show the behaviour of the liquid behind the disturbing element with increasing distance from the disturbing element.

In the described projections, especially in the visualization of the flow corresponding to the Reynolds number ≅ 100,000, the uniformity of the flow velocity distribution in the cross-section of the pipeline is observed, which is from a distance equal to six nominal diameters from the outflow of the medium measured from the 90° bend. Contours of velocity distribution are arranged evenly in a circular pattern. It means that the flow velocity is homogenised in the entire cross-sectional plane of the pipeline.

This was presented in the form of a uniform colour in the central part of the pipeline and proves a clear stabilization of the velocity profile for different Reynolds numbers. The circularity of zones with the same velocity is clear. It can be concluded that at a distance of twelve nominal diameters from the bend, which is the disturbing element, it is possible to measure the stream flow. Performing flow measurements at the distance makes it very likely that the metrological parameters declared by the manufacturer of the measuring device will be maintained.

It can be concluded that at a distance of twelve nominal diameters from the bend, which is the disturbing element, for Re > 70,000, it is possible to measure the stream flow. Performing flow measurements at this distance makes it very likely that the metrological parameters declared by the manufacturer of the measuring device will be maintained.

After calculating the integrals from the surfaces obtained as a result of measurements of the 3D velocity distribution obtained by the laser anemometry method, the results were v_avg_ = 2.025 m/s for the flow, where Re ≅ 100,000 and v_avg_ = 1.408 m/s for the flow, where Re ≅ 70,000.

In the velocity profiles for the 12D plane behind the 90° bend, the minimum and maximum velocities are clearly noticeable, which proves the non-homogeneous flow.

It is also noticeable that the profiles are similar in terms of quality. The profiles shown in Figure 9 and Figure 10 intersect in the pipeline axis, which proves the correctness of the measurement.

In the above figures showing the two-dimensional velocity profiles, it can be seen that both, in a flow with a Reynolds number of ≅ 100,000 and in a flow with Reynolds number of ≅ 70,000, the shapes of the profiles at individual angles are similar and coincide.

**Figure 25:**
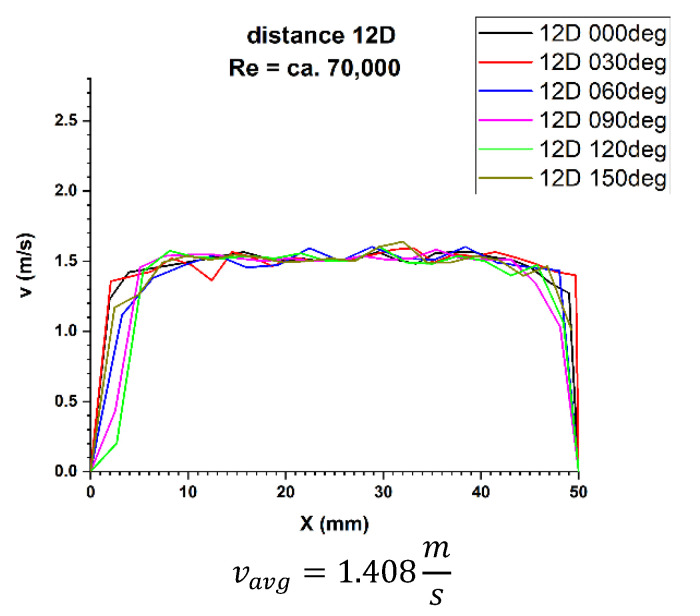
Velocity profiles at a distance 12D from the disturbance with marked cross-sections for Re ≅ 70,000-The LDA measurement taken in the ultrasound wave path.

**Figure 26:**
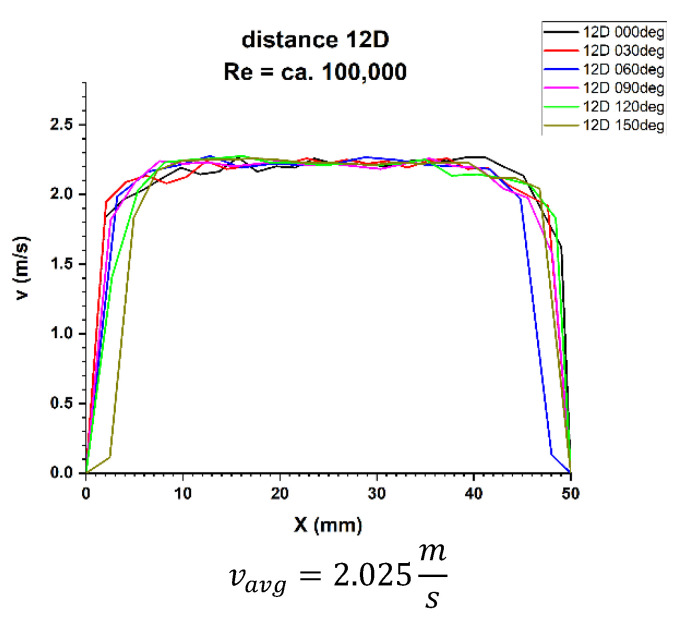
Velocity profiles at a distance 12D from the disturbance with marked cross-sections for Re ≅ 100,000-The LDA measurement taken in the ultrasound wave path.

In both cases (Figure 25 and Figure 26) a flattening of the profile in the central part of the pipeline was noticed.

Based on the obtained measurement results (Table 9 and Table 10), it can be concluded that the profiles are stabilized. It can be seen from the test results that for the Reynolds number 100,000 and the plane located at a distance of 12D behind the 90° bend, there are two installation angles of the ultrasound heads where the errors of the direct measurements are the smallest. For angles of 60° and 240° it is 0.4%. There is also a noticeable difference Δv_avg_ equal 0.8% between the minimum and maximum measurements made behind the 90° bend the 12D plane. The mean correction factor K^*^_avg_ is 1%, and when multiplying the obtained results by a coefficient reflecting Δv_avg_, which is 1.008, the measurements made at all angles do not exceed the maximum permissible error (MPE) of the ultrasonic flowmeters.

Therefore, it was checked whether the measurements taken in the 12D plane, with Reynolds number ≅ 100,000, should be provided with an average correction factor of 1.010 in order to minimize the uncertainty of the measurements.

## 5. Summary

The research carried out allowed us to draw the following conclusions:

The lengths of straight sections necessary for flow measurements behind disturbance, which are within the maximum permissible error (MPE) of the flow meter, more than 15 pipeline diameters are too large.

In the case of ultrasonic flow meters, the tests carried out have shown that already at a distance of 8 pipeline diameters from the disturbance, it is possible to perform a correct measurement with a maximum permissible error (MPE) less than 2% of the indicated flow stream value.

Results of researches carried out for 8D distance have been illustrated in Table 5 and Table 6.

For distances smaller than eight pipeline diameters, there are places on the circuit of the pipeline, in a given cross-section, where the error of the flow stream measurement is the smallest–behind the 90° bend these are respectively 60° and 240°.

The standard deviations of the mean value of the correction factor K* in Table 2, Table 3, Table 4, Table 5, Table 6, Table 7, Table 8, Table 9 and Table 10 change from 2.7% for a distance of 0D (nominal diameters) from the disturbance to 0.5% for distances from the disturbance greater than 8D (nominal diameters). For these values, with the assumption that the uncertainty of the pipeline diameter is to be ignored and the velocity uncertainty is determined by the type B method from the ultrasonic flow meter limit error Δg = ±2%, the relative standard uncertainty of the measured flow rate can be estimated. For distances of 0D (nominal diameters) from the disturbance it is about 3% and for distances larger than 8D (nominal diameters) it is about 1.3%.

Measurements have shown that it is possible to measure the flow stream in the entire pipeline circuit with an interval of 30° and taking into account the average value of the K^*^ coefficient. The averaged value of this coefficient in the range from 0D to 7D is in the range (1.108–1.026), therefore, for each distance from the disturbance, a different value of this coefficient should be taken into account. It can also be noticed that using the value $K_{avg}^{*}=\frac{\left(1.108+1.026\right)}{2}\approx 1.07$, for each distance from the disturbance in the range 0D–7D, we will not make a greater than 4% error in measuring the flow stream.

It is also worth noting that the values of the measured velocity (volume stream) after the disturbance are always smaller than in the long straight section-they have a systematic error. This phenomenon can be explained by the fact that in the case of a disturbed distribution of the flow velocity behind the 90° bend, the ultrasonic wave radius sent from the transmitter to the receiver passes through the velocity distributions in places where it reaches a low value, always lower than the average value, therefore the resultant velocity will always be lower from the mean value indicated by the calibration flow meter.

## Figures and Tables

**Table 1:** Characteristic values of measuring devices.

No. on Drawing	Device	Label	Model	Specification	Maximum Permissible Error (MPE)
4	Electromagnetic flow meter	E+H	Promag 53W	DN50, current loop 4-20 mA	±(0.2% × measured value)
5	ISA orifice plate	---	Annular Chamber Pressure Tapping	D = 35.71mm D = 50.00 mm C = 0.602	±(2% × measured value)
9	Data logger	Lumel	KD7	Inputs of current loop 4-20 mA	±0.25% × measurement range
11	Ultrasonic flow meter	Micronics	Portaflow PF330	Transit time measurement, Clamp-on sensors	0.5% to 2% of speed reading for v > 0.2 m/s
13	Ultrasonic flow meter	E+H	Prosonic Flow 93T	Transit Time measurement, Clamp-on sensors	0.5% to 2% of speed reading for v > 0.3 m/s for Re > 10,000
14	Laser Doppler anemometer	Dantec	One-channel laser Doppler anemometer	power of laser: 10 mW light wavelength: 632.8 nm-red light focal length: 160 mm measuring volume: 75 μm × 630 μm	---

**Table 2:** Detailed specification of measurement parameters.

Name of Parameter	Parameter
Nominal diameter of the research pipeline	DN 50
Distance between sensors	ca. 91mm
Velocities	w1 = 1.418 m/s for Re ca. 70,000 w2 = 2.04 m/s for Re ca. 100,000
The ultrasound wave path	2V
Sampling interval	5s

**Table 3:** Summary of the results of ultrasonic measurements for the 0D plane from the disturbing element as a function of the mounting angle of the ultrasonic flow meter sensors, Re ≅ 70,000.

0D, Re ≅ 70,000												
angle φ in °	0	30	60	90	120	150	180	210	240	270	300	330
***v behind bend*** m/s												
v_avg_ 93T ± 2%	1.237	1.290	1.313	1.302	1.273	1.273	1.249	1.287	1.305	1.302	1.277	1.248
q_avg_ 93T ±2%	8.743	9.119	9.284	9.202	8.998	8.998	8.826	9.099	9.224	9.200	9.024	8.819
***v before bend*** m/s												
v_avg_ PF330 ± 2%	1.418											
q_avg_ PF330 ± 2%	10.022											
**Re before bend PF330**	**69,819**											
**correction factor**												
K^*^ [v_before avg_/v_behind avg_] ***PF330/ PS93T***	**1.146**	**1.099**	**1.079**	**1.089**	**1.114**	**1.114**	**1.135**	**1.101**	**1.086**	**1.089**	**1.111**	**1.136**
K^*^_avg_	**1.108 ± 0.022**											

**Table 4:** Summary of the results of ultrasonic measurements for the 0D plane from the disturbing element as a function of the mounting angle of the ultrasonic flow meter sensors, Re ≅ 100,000.

0D, Re ≅ 100,000												
angle φ in °	0	30	60	90	120	150	180	210	240	270	300	330
***v behind bend*** m/s												
v_avg_ 93T ± 2%	1.797	1.852	1.932	1.862	1.805	1.788	1.792	1.852	1.932	1.862	1.810	1.830
q_avg_ 93T ± 2%	12.706	13.090	13.654	13.161	12.759	12.641	12.670	13.090	13.654	13.161	12.794	12.936
***v before bend*** m/s												
v_avg_ PF330 ± 2%	2.040											
q_avg_ PF330 ± 2%	14.420											
**Re before bend PF330**	**100,462**											
**correction factor**												
K^*^ [v_before avg_/v_behind avg_] ***PF330/ PS93T***	**1.146**	**1.102**	**1.056**	**1.096**	**1.130**	**1.141**	**1.138**	**1.102**	**1.056**	**1.096**	**1.127**	**1.115**
K^*^_avg_	**1.108 ± 0.030**											

**Table 5:** Summary of the results of ultrasonic measurements for the 6D plane from the disturbing element as a function of the mounting angle of the ultrasonic flow meter sensors, Re ≅ 70,000.

6D, Re ≅ 70,000												
angle φ in °	0	30	60	90	120	150	180	210	240	270	300	330
***v behind bend*** m/s												
v_avg_ 93T ± 2%	1.355	1.358	1.370	1.349	1.340	1.345	1.355	1.371	1.374	1.364	1.345	1.335
q_avg_ 93T ± 2%	9.576	9.602	9.685	9.536	9.471	9.506	9.576	9.688	9.710	9.638	9.511	9.435
***v before bend*** m/s												
v_avg_ PF330 ± 2%	1.418											
q_avg_ PF330 ± 2%	10.022											
**Re before bend PF330**	**69,819**											
**correction factor**												
K^*^ [v_before avg_/v_behind avg_] ***PF330/ PS93T***	**1.047**	**1.044**	**1.035**	**1.051**	**1.058**	**1.054**	**1.047**	**1.034**	**1.032**	**1.040**	**1.054**	**1.062**
K^*^_avg_	**1.046 ±0.010**											

**Table 6:** Summary of the results of ultrasonic measurements for the 6D plane from the disturbing element as a function of the mounting angle of the ultrasonic flow meter sensors, Re ≅ 100,000.

6D, Re ≅ 100,000												
angle φ in °	0	30	60	90	120	150	180	210	240	270	300	330
***v behind bend*** m/s												
v_avg_ 93T ± 2%	1.972	1.985	1.996	1.963	1.962	1.965	1.981	1.992	1.996	1.985	1.969	1.974
q_avg_ 93T ± 2%	13.939	14.032	14.108	13.872	13.869	13.889	14.006	14.079	14.110	14.029	13.921	13.957
***v before bend*** m/s												
v_avg_ PF330 ± 2%	2.040											
q_avg_ PF330 ± 2%	14.420											
**Re before bend PF330**	**100,462**											
**correction factor**												
K^*^ [v_before avg_/v_behind avg_] ***PF330/ PS93T***	**1.034**	**1.028**	**1.022**	**1.039**	**1.040**	**1.038**	**1.030**	**1.024**	**1.022**	**1.028**	**1.036**	**1.033**
K^*^_avg_	**1.031 ± 0.007**											

**Table 7:** Summary of the results of ultrasonic measurements for the 8D plane from the disturbing element as a function of the mounting angle of the ultrasonic flow meter heads, Re ≅ 70,000.

8D, Re ≅ 70,000												
angle φ in °	0	30	60	90	120	150	180	210	240	270	300	330
***v behind bend*** m/s												
v_avg_ 93T ± 2%	1.403	1.403	1.407	1.390	1.394	1.394	1.397	1.396	1.405	1.395	1.389	1.386
q_avg_ 93T ± 2%	9.917	9.921	9.946	9.824	9.853	9.853	9.877	9.870	9.928	9.860	9.818	9.795
***v before bend*** m/s												
v_avg_ PF330 ± 2%	1.418											
q_avg_ PF330 ± 2%	10.022											
**Re before bend PF330**	**69,819**											
**correction factor**												
K^*^ [v_before avg_/v_behind avg_] ***PF330/ PS93T***	**1.011**	**1.010**	**1.008**	**1.020**	**1.017**	**1.017**	**1.015**	**1.015**	**1.009**	**1.016**	**1.021**	**1.023**
K^*^_avg_	**1.015 ± 0.005**											

**Table 8:** Summary of the results of ultrasonic measurements for the 8D plane from the disturbing element as a function of the mounting angle of the ultrasonic flow meter heads, Re ≅ 100,000.

8D, Re ≅ 100,000												
angle φ in °	0	30	60	90	120	150	180	210	240	270	300	330
***v behind bend*** m/s												
v_avg_ 93T ± 2%	2.003	2.005	2.011	1.991	1.990	1.995	2.001	2.006	2.010	2.003	1.991	1.994
q_avg_ 93T ± 2%	14.158	14.173	14.217	14.072	14.066	14.102	14.144	14.183	14.208	14.158	14.074	14.095
***v before bend*** m/s												
v_avg_ PF330 ± 2%	2.040											
q_avg_ PF330 ± 2%	14.420											
**Re before bend PF330**	**100,462**											
**correction factor**												
K^*^ [v_before avg_/v_behind avg_] ***PF330/ PS93T***	**1.018**	**1.017**	**1.014**	**1.025**	**1.025**	**1.023**	**1.019**	**1.017**	**1.015**	**1.018**	**1.025**	**1.023**
K^*^_avg_	**1.020 ± 0.004**											

**Table 9:** Summary of the results of ultrasonic measurements for the 12D plane from the disturbing element as a function of the mounting angle of the ultrasonic flow meter heads, Re ≅ 70,000.

12D, Re ≅ 70,000												
angle φ in °	0	30	60	90	120	150	180	210	240	270	300	330
***v behind bend*** m/s												
v_avg_ 93T ± 2%	1.410	1.413	1.415	1.410	1.401	1.401	1.409	1.409	1.412	1.404	1.403	1.409
q_avg_ 93T ± 2%	9.964	9.987	10.005	9.964	9.903	9.903	9.963	9.960	9.980	9.921	9.915	9.958
***v before bend*** m/s												
v_avg_ PF330 ± 2%	1.418											
q_avg_ PF330 ± 2%	10.022											
**Re before bend PF330**	**69,819**											
**correction factor**												
K^*^ [v_before avg_/v_behind avg_] ***PF330/PS93T***	**1.006**	**1.003**	**1.002**	**1.006**	**1.012**	**1.012**	**1.006**	**1.006**	**1.004**	**1.010**	**1.011**	**1.006**
K^*^_avg_	**1.007 ± 0.004**											

**Table 10:** Summary of the results of ultrasonic measurements for the 12D plane from the disturbing element as a function of the mounting angle of the ultrasonic flow meter heads, Re ≅ 100,000.

12D, Re ≅ 100,000												
angle φ in °	0	30	60	90	120	150	180	210	240	270	300	330
***v behind bend*** m/s												
v_avg_ 93T ± 2%	2.027	2.028	2.032	2.025	2.019	2.019	2.026	2.033	2.033	2.020	2.016	2.027
q_avg_ 93T ± 2%	14.327	14.333	14.363	14.315	14.270	14.270	14.318	14.372	14.368	14.279	14.252	14.327
***v before bend*** m/s												
v_avg_ PF330 ± 2%	2.04											
q_avg_ PF330 ± 2%	14.42											
**Re before bend PF330**	**100,462**											
**correction factor**												
K^*^ [v_before avg_/v_behind avg_] ***PF330/PS93T***	**1.006**	**1.006**	**1.004**	**1.007**	**1.011**	**1.011**	**1.007**	**1.003**	**1.004**	**1.010**	**1.012**	**1.007**
K^*^_avg_	**1.007 ± 0.003**											

## Data Availability

All data are integral part of the correspondent author’s PhD thesis.

## Abbreviations

**List of Symbols**	
D	nominal diameter of the pipeline
R	bending radius of 90° bend
Re	Reynolds number
$Re_{MAX}$	maximum Reynolds number $q_{v}$—volume stream
$q_{v\;MAX}$	maximum volume stream
v	flow velocity
$v_{MAX}$	maximum flow velocity
$v_{avg}$	average flow velocity
